# Supplementary figures and images for: The impact of three thioxothiazolidin compounds on trehalase activity and development of Spodoptera frugiperda larvae
Source: PeerJ. 2024 Oct 9;12:e18233. doi: 10.7717/peerj.18233 (PMC11470766; doi:10.7717/peerj.18233)

## Slide 1
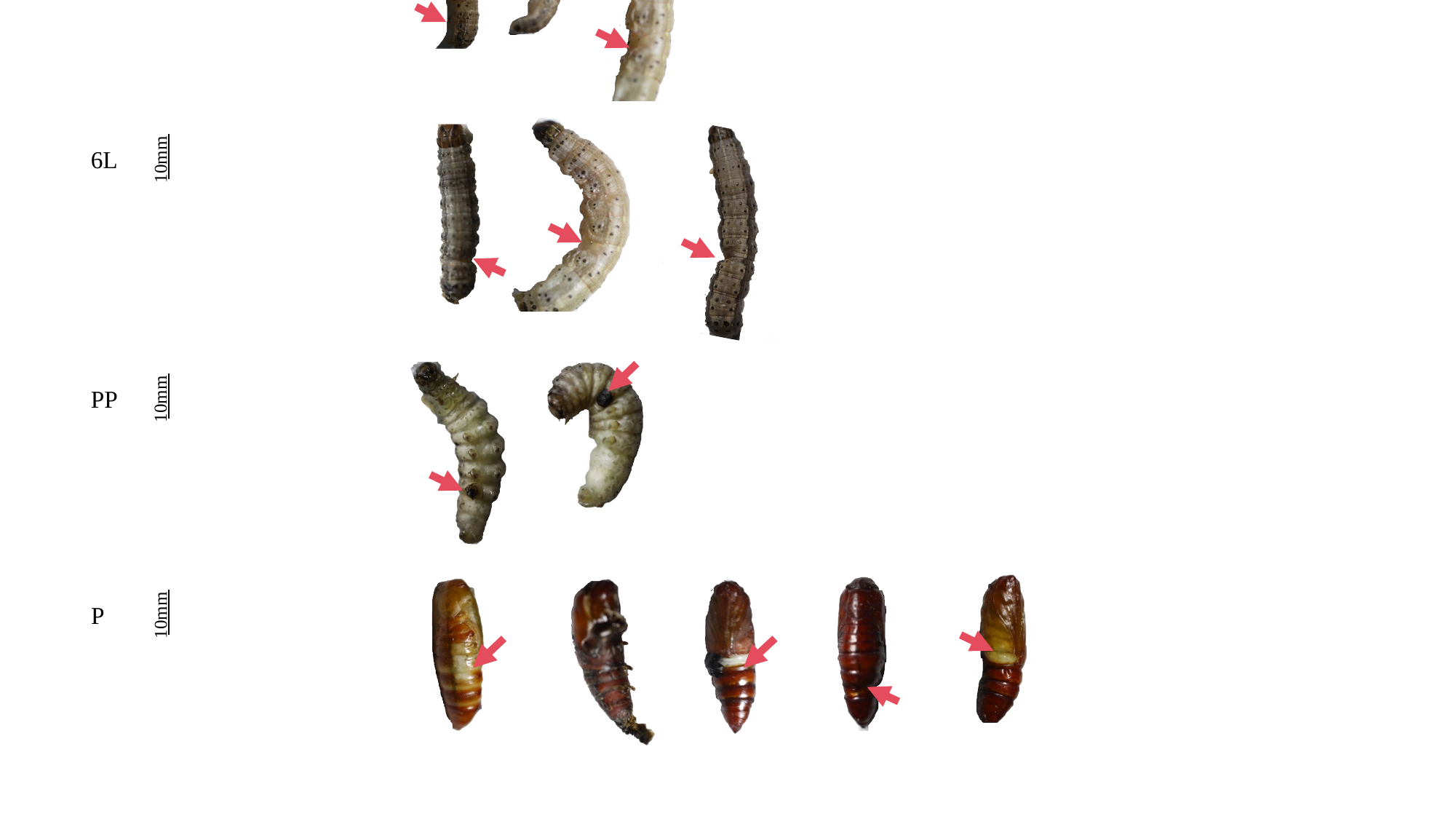

正常表型
异常表型
4L
5mm
5L
10mm
6L
10mm
PP
10mm
P
10mm

Supplement: Supplemental Information 1 [file peerj-12-18233-s001.zip › peerj-101514-phenotype/Picture of phenotypes.pptx]

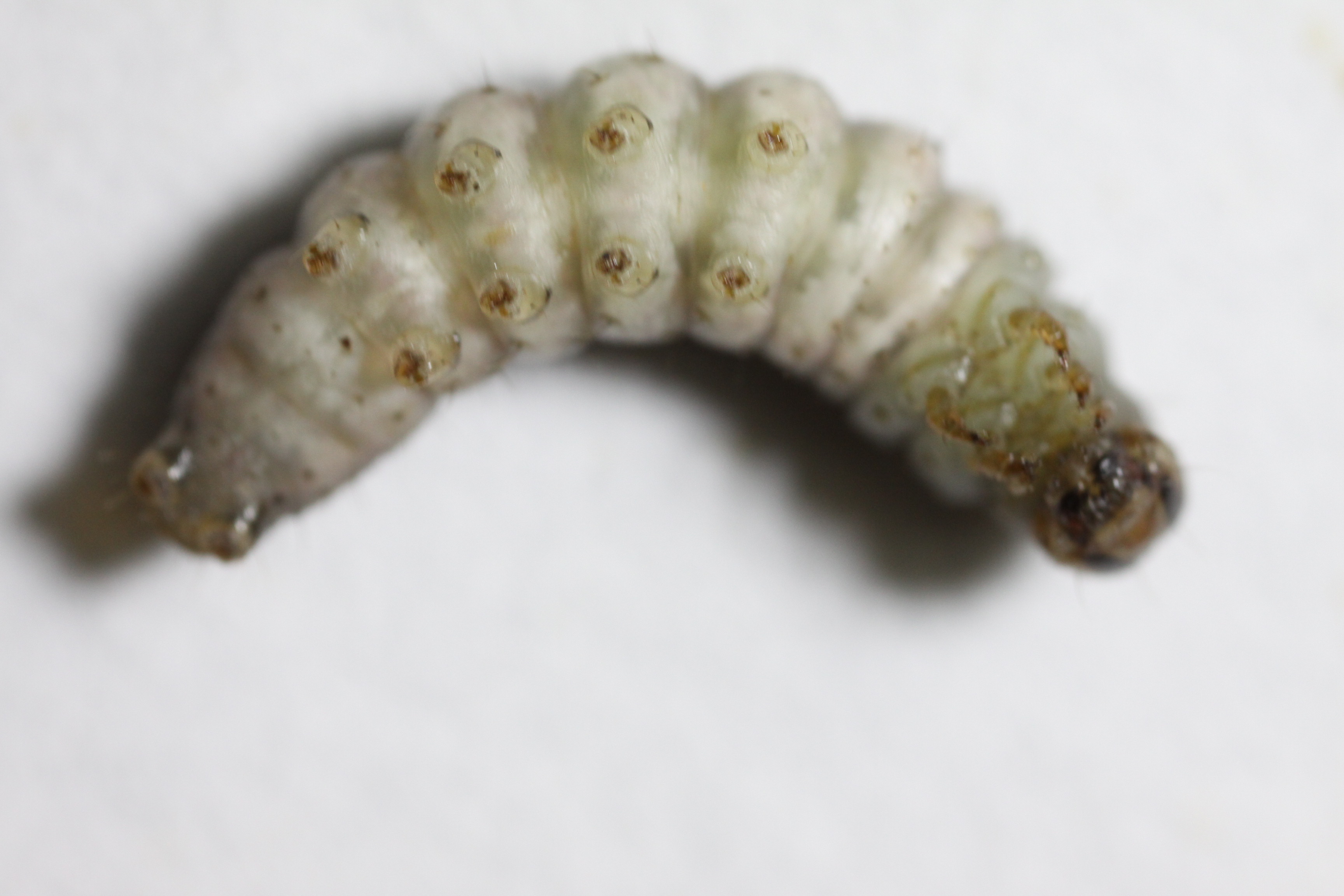

Supplement: Supplemental Information 1 [file peerj-12-18233-s001.zip › peerj-101514-phenotype/Picture of the normal phenotype at the prepupal stage.JPG]

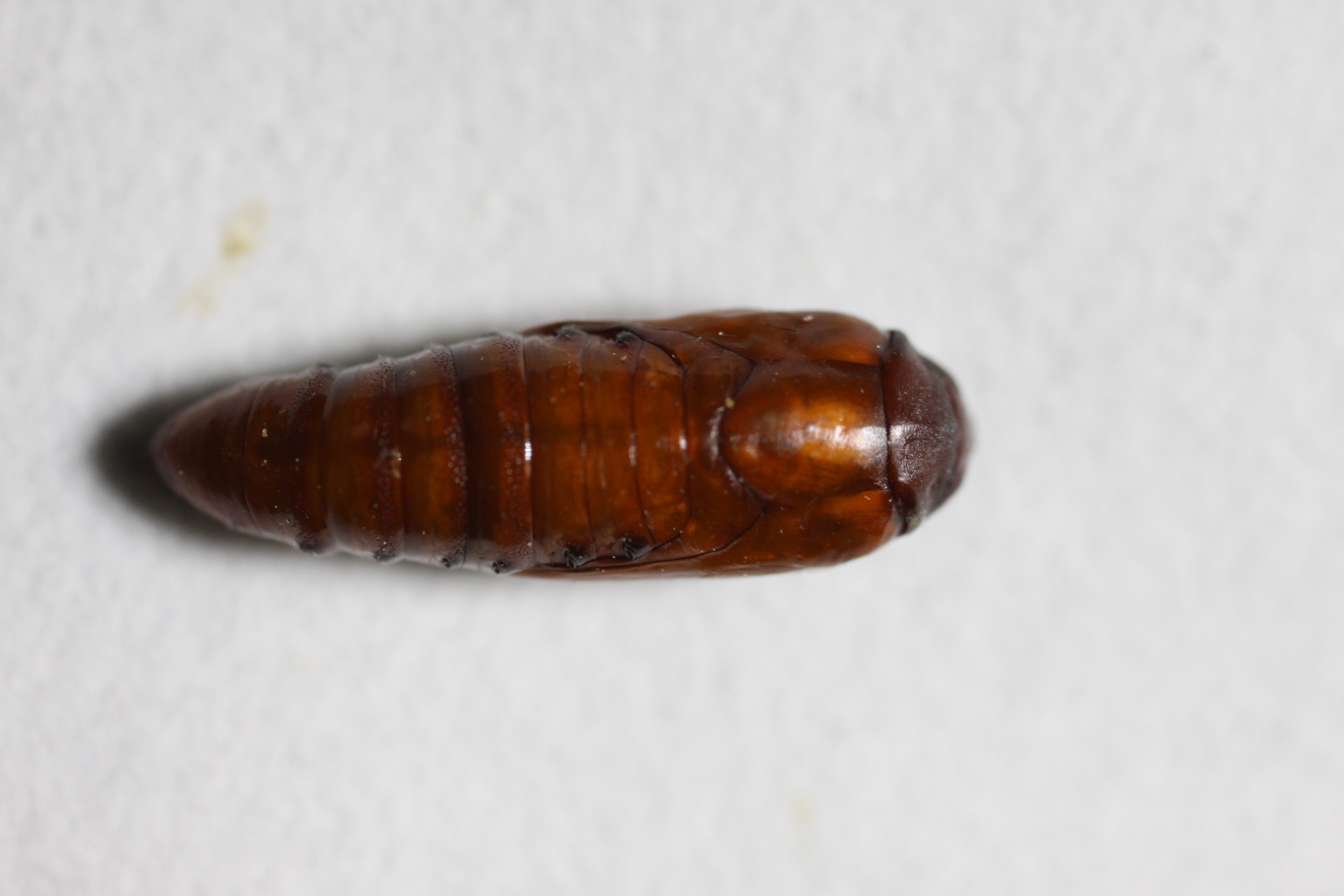

Supplement: Supplemental Information 1 [file peerj-12-18233-s001.zip › peerj-101514-phenotype/Picture of the normal phenotype at the pupal stage.JPG]

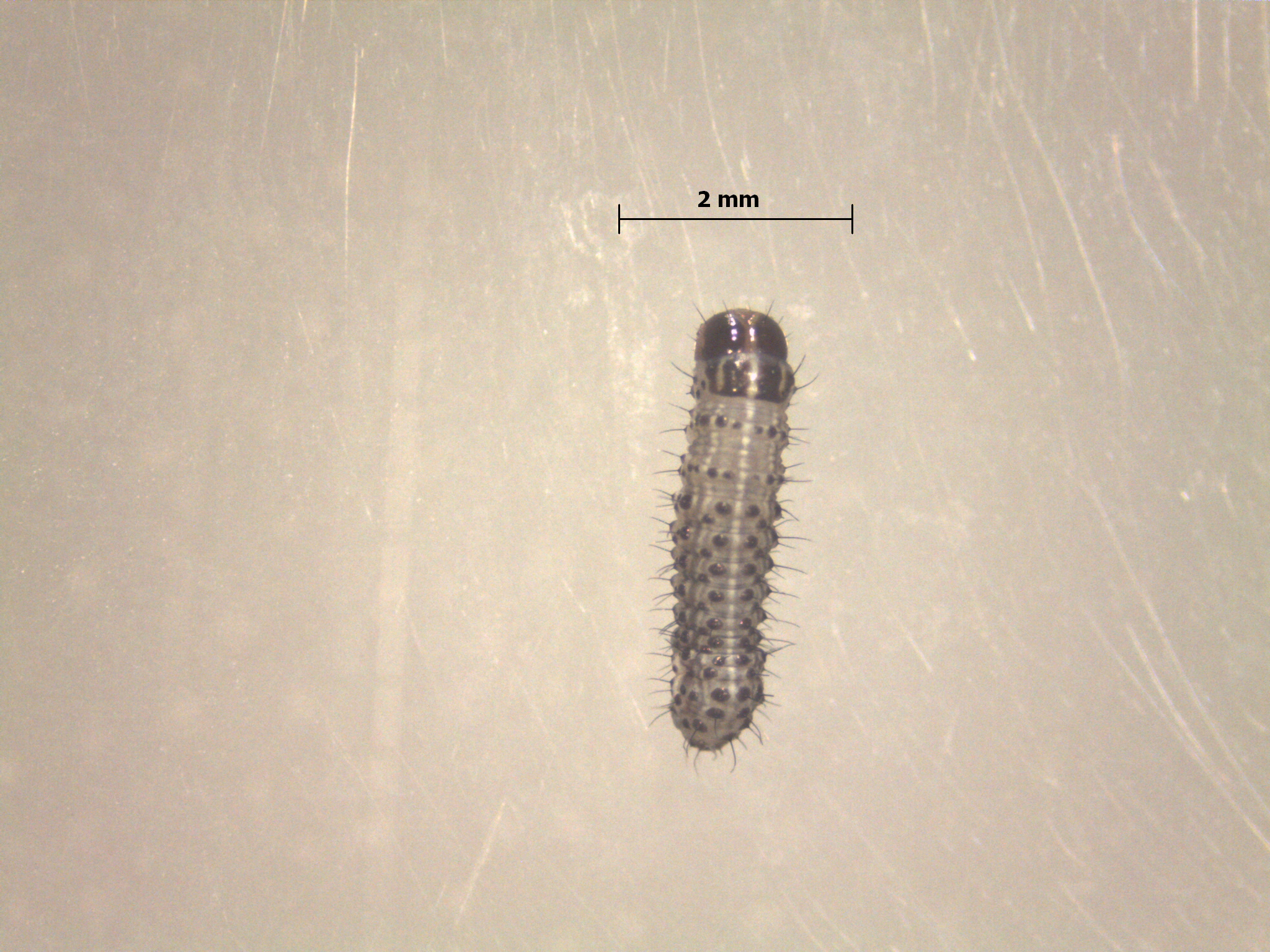

Supplement: Supplemental Information 1 [file peerj-12-18233-s001.zip › peerj-101514-phenotype/Pictures of normal phenotypes of 4th instar larvae.jpg]

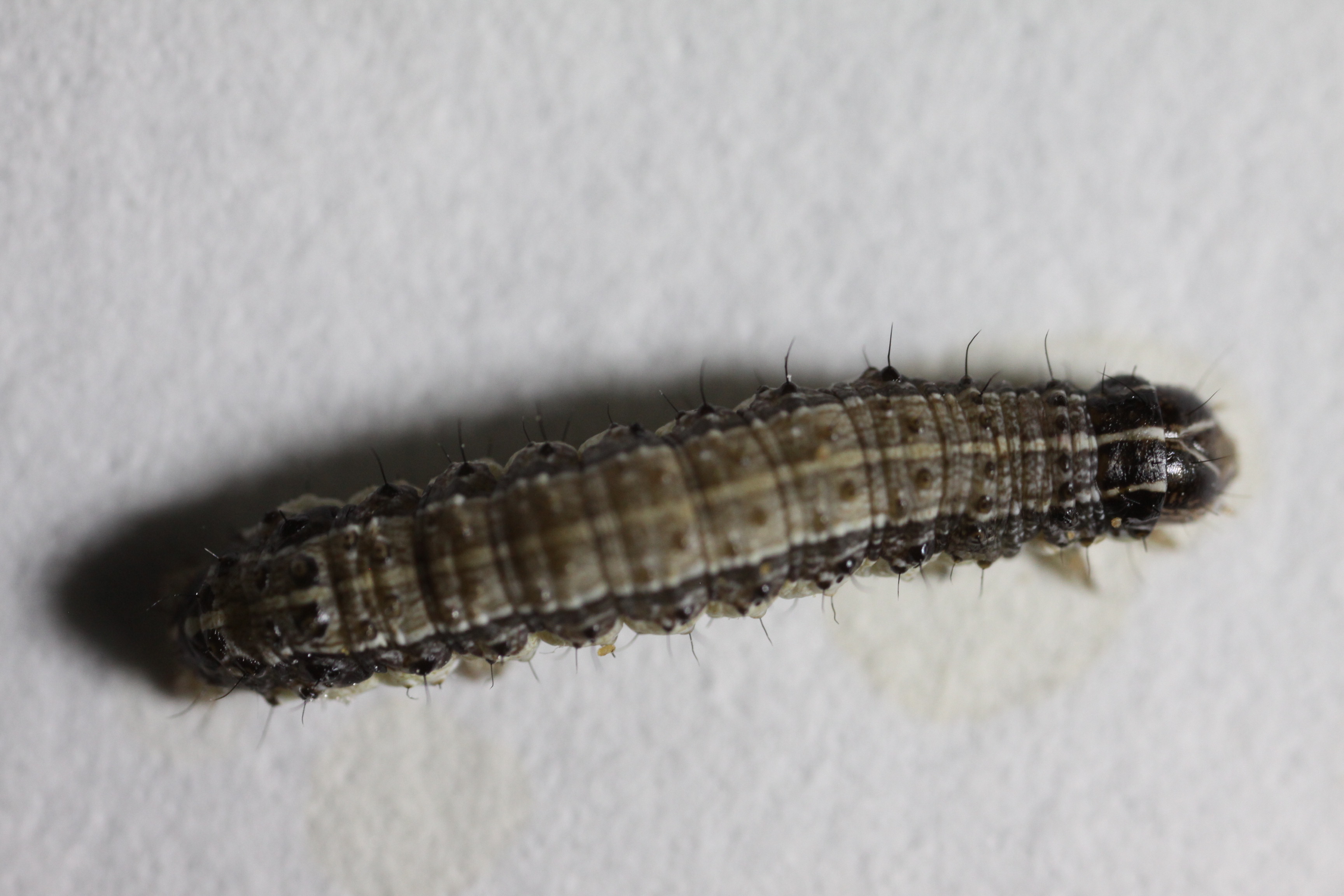

Supplement: Supplemental Information 1 [file peerj-12-18233-s001.zip › peerj-101514-phenotype/Pictures of normal phenotypes of 5th instar larvae.JPG]

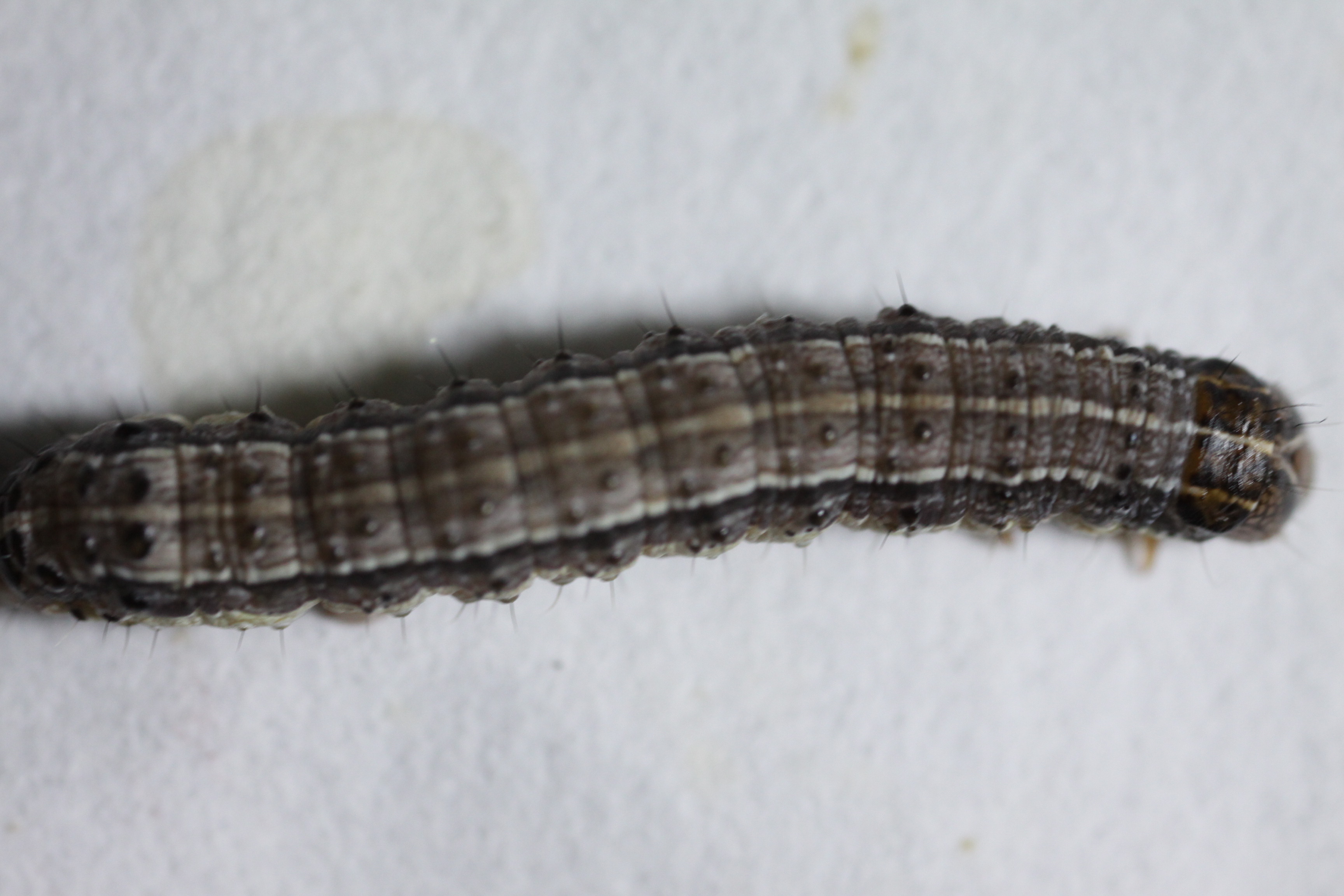

Supplement: Supplemental Information 1 [file peerj-12-18233-s001.zip › peerj-101514-phenotype/Pictures of normal phenotypes of 6th instar larvae.JPG]
